# Case Report: HCV-triggered porphyria cutanea tarda in a patient with SEC23B-mutated congenital dyserythropoietic anemia type II

**DOI:** 10.3389/fmed.2026.1817500

**Published:** 2026-07-13

**Authors:** Xianghong Jin, Yixuan Li, Yaping Liu, Xianyong Jiang, Junling Zhuang, Min Shen, Miao Chen

**Affiliations:** 1 Department of Rare Diseases, Peking Union Medical College Hospital, Chinese Academy of Medical Sciences & Peking Union Medical College, Beijing, China; 2 Center for Rare Diseases, Peking Union Medical College Hospital, Chinese Academy of Medical Sciences & Peking Union Medical College, Beijing, China; 3 Department of Hematology, Peking Union Medical College Hospital, Chinese Academy of Medical Sciences & Peking Union Medical College, Beijing, China

**Keywords:** congenital dyserythropoietic anemia type II, hepatitis C virus, iron overload, porphyria cutanea tarda, SEC23B mutation

## Abstract

Porphyria cutanea tarda (PCT) is a hepatic porphyria often triggered by hepatitis C virus (HCV) infection, iron overload, or environmental exposure. Congenital dyserythropoietic anemia type II (CDA II) caused by SEC23B mutations leads to ineffective erythropoiesis and secondary iron accumulation. We report a 34-year-old man with genetically confirmed SEC23B-mutated CDA II who developed photosensitive bullae associated with chronic HCV genotype 1b infection, hyperbilirubinemia, and severe iron overload. Urinary porphyrins were positive, and skin biopsy showed subepidermal bullae with PAS-positive deposits, supporting the diagnosis of PCT. After unsuccessful therapy with hydroxychloroquine, treatment with sofosbuvir/velpatasvir achieved sustained virologic response, resolution of skin lesions, and marked ferritin reduction. This case illustrates that viral and metabolic stressors can trigger PCT in CDA II patients with possible hepatic involvement, underscoring the importance of recognizing combined genetic and infectious factors in rare hematologic disorders.

## Case presentation

**Figure 1:**
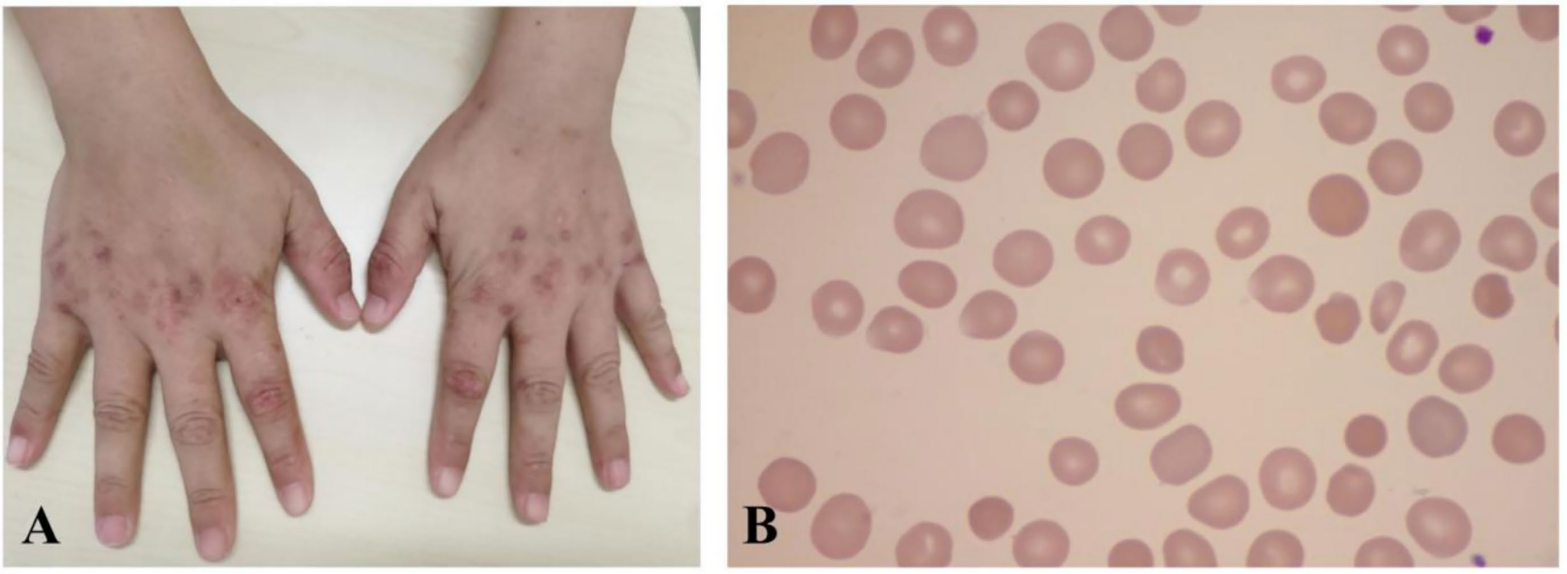
Clinical phenotype and peripheral blood morphology. **(A)** Photodistributed vesiculobullous and erosive lesions with crusting and post-inflammatory hyperpigmentation on the dorsal hands, consistent with porphyria cutanea tarda. **(B)** Peripheral blood smear showing anisopoikilocytosis with numerous stomatocytes, consistent with congenital dyserythropoietic anemia type II.

A 34-year-old male construction worker presented in October 2023 with recurrent vesiculobullous eruptions on sun-exposed skin (dorsal hands, neck, and face), exacerbated by occupational asphalt exposure and UV irradiation. Cutaneous examination revealed hyperpigmented atrophic scars, milia, and subepidermal bullae with dermal festooning (Figure 1A). Hepatosplenomegaly was noted on abdominal palpation. His medical history included lifelong hemolytic anemia and intermittent jaundice, requiring occasional transfusions.

Laboratory findings demonstrated normocytic anemia (hemoglobin 120 g/L, MCV 90 fL), unconjugated hyperbilirubinemia (total bilirubin 71.7 μmol/L, direct bilirubin 10.2 μmol/L), and elevated liver enzymes (ALT 57 U/L, GGT 64 U/L). Peripheral blood smear revealed anisocytosis and spherocytes (Figure 1B). Iron studies confirmed severe overload (ferritin 1,201 ng/mL, transferrin saturation 94.7%). Plasma free erythrocyte protoporphyrin was increased to 13.3 μg/g Hb, which was considered attributable to the underlying dyserythropoiesis associated with CDA II. Urinary porphyrins were positive, while fecal porphyrins could not be assessed. Fractionated urinary porphyrin analysis was not available. Active HCV genotype 1b infection was confirmed (HCV RNA 9.42 × 10^5^ IU/mL). Tests for HIV and hepatitis B were negative. Besides, skin biopsy showed subepidermal bullae with perivascular hyaline deposits (PAS-positive), consistent with PCT.

**Figure 2:**
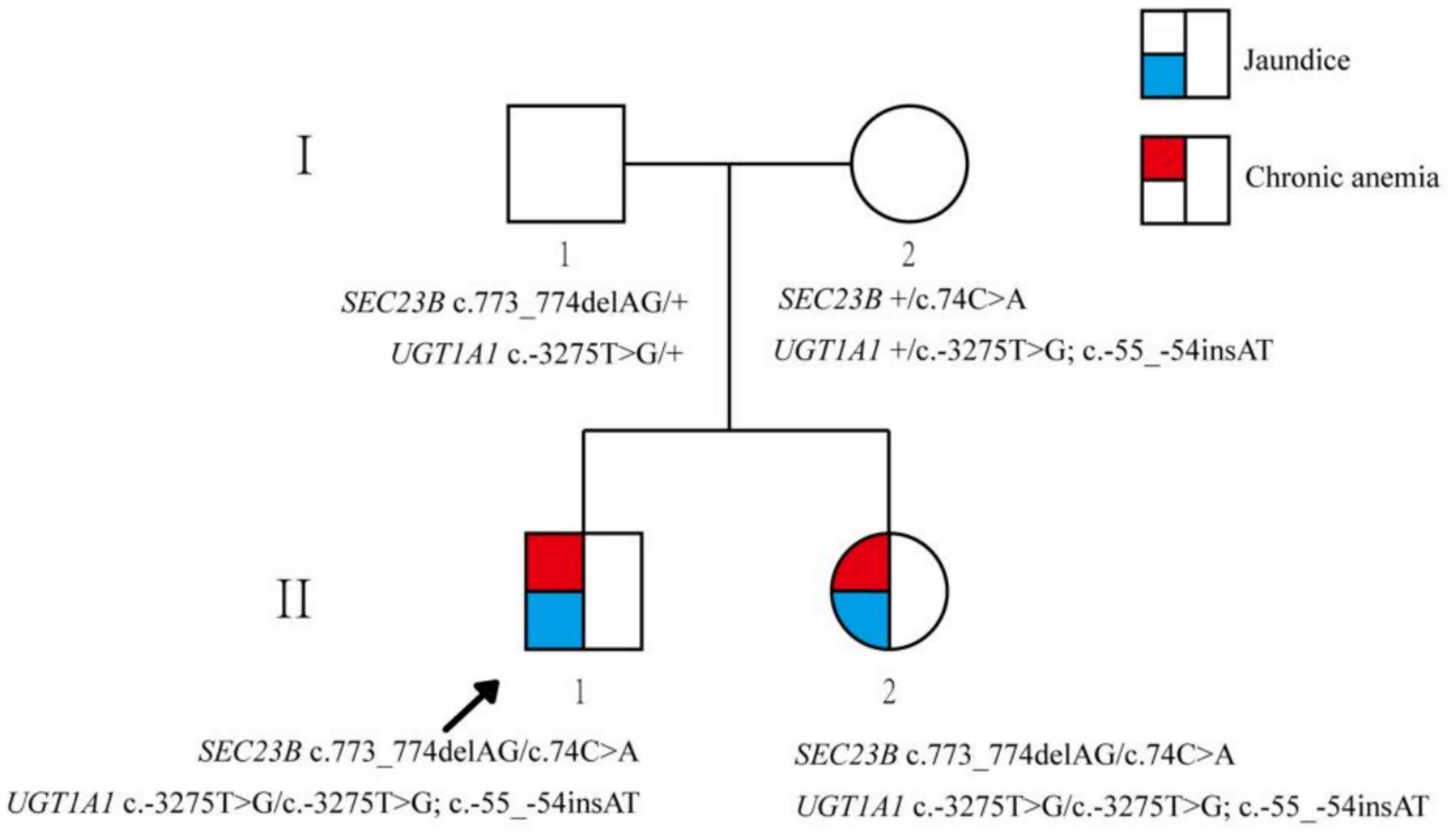
Genetic findings and family segregation analysis. Pedigree analysis showing compound heterozygous SEC23B variants in the proband, with segregation of variants within the family. Coexisting UGT1A1 variants are also indicated.

Whole-exome sequencing identified compound heterozygous SEC23B variants: c.773_774delAG (p.Gln258Argfs49), classified as pathogenic, and c.74C > A (p.Pro25His), likely pathogenic. Both were inherited from heterozygous carrier parents. The patient also carried a homozygous UGT1A1 promoter polymorphism (c.-3275 T > G), compatible with Gilbert’s syndrome. Family segregation confirmed asymptomatic carrier status in parents and hemolysis in a compound heterozygous sister (Figure 2).

Abdominal ultrasound demonstrated mild hepatomegaly (right lobe 16.6 cm) and splenomegaly (length 20 cm) with slightly heterogeneous liver echogenicity. Liver elastography yielded 7.4 kPa, suggesting mild fibrosis but no cirrhosis. Other organs were unremarkable.

**Figure 3:**
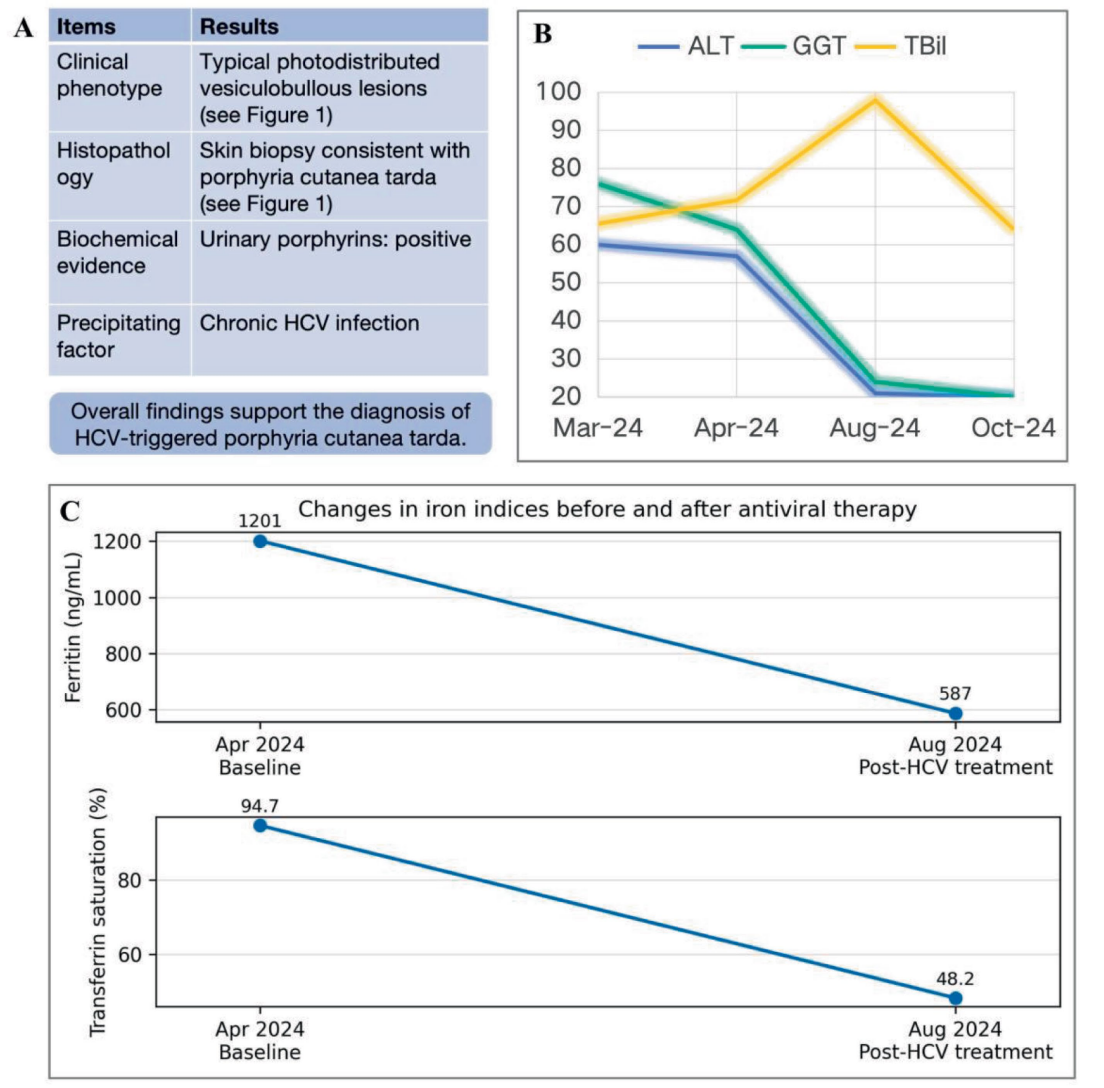
Diagnostic evaluation and laboratory findings. **(A)** Summary of clinical, histopathological, and biochemical findings supporting the diagnosis of porphyria cutanea tarda. **(B)** Longitudinal changes in liver enzymes and bilirubin levels. **(C)** Changes in iron indices before and after antiviral therapy, showing a marked reduction in ferritin and transferrin saturation.

The patient was diagnosed with HCV-induced PCT, CDA II, and Gilbert’s syndrome. Initial therapy with hydroxychloroquine (100 mg twice weekly) and nicotinamide produced no improvement. In May 2024, direct-acting antiviral therapy with sofosbuvir/velpatasvir (400/100 mg once daily) was started for 12 weeks. The patient tolerated treatment well, and HCV RNA became undetectable at week 12. Skin lesions completely resolved, and ferritin decreased to 587 ng/mL within 3 months. At six-month follow-up, he remained asymptomatic with normal liver enzymes and stable hemoglobin. The diagnostic findings and longitudinal laboratory changes are summarized in Figure 3.

## Discussion

**Figure 4:**
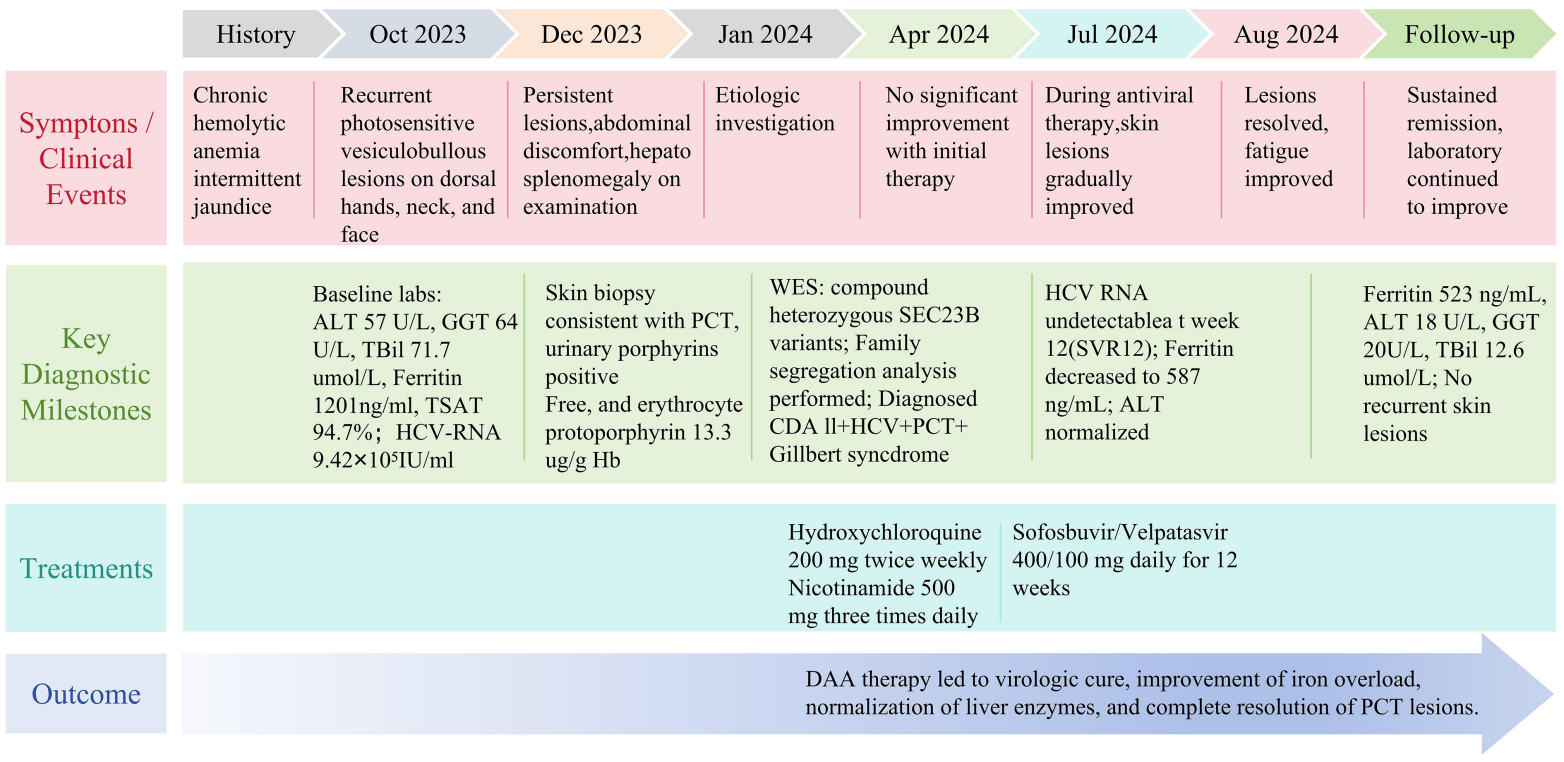
Clinical timeline of symptoms, diagnostic work-up, treatment, and outcome. Timeline summarizing clinical presentation, diagnostic evaluation, treatment interventions, and outcomes. Antiviral therapy led to virologic clearance, improvement of iron overload, normalization of liver function, and resolution of cutaneous lesions.

This case represents the first report of hepatitis C virus (HCV)-triggered porphyria cutanea tarda (PCT) occurring in a patient with genetically confirmed SEC23B-mutated congenital dyserythropoietic anemia type II (CDA II). The overall clinical course, including symptoms, diagnostic evaluation, treatment, and outcomes, is illustrated in Figure 4. The coexistence of these rare conditions suggested that the genetic, viral, and environmental factors may converge on shared hepatic pathways of iron and oxidative metabolism (Supplementary Figure). The mechanistic model is based on established molecular knowledge rather than experimental data from this patient and should be interpreted as a biologically plausible and hypothesis-generating explanation.

In CDA II, loss-of-function variants in SEC23B impair COPII-mediated trafficking from the endoplasmic reticulum (ER) to the Golgi apparatus in erythroid precursors, leading to *α*-globin retention, ineffective erythropoiesis, and chronic hemolysis (1, 2). Persistent hemolysis suppresses hepatic hepcidin, resulting in systemic iron overload and hepatic iron deposition. Excess iron promotes oxidative stress through Fenton reactions, rendering hepatocytes more susceptible to secondary insults such as viral infection or xenobiotic activation. In this patient, a ferritin concentration exceeding 1,200 ng/mL suggested severe systemic iron overload, possibly with hepatic involvement, which could have primed the liver for porphyrinogenic stress.

HCV infection is a well-recognized precipitant of PCT (3–5). Viral proteins, particularly NS5A, can generate reactive oxygen species that may inhibit uroporphyrinogen decarboxylase (UROD) activity, leading to accumulation of uroporphyrin I and heptacarboxylporphyrin. Concurrently, HCV may down-regulates hepcidin, further enhancing iron absorption and perpetuating oxidative injury. Thus, in the context of CDA II-related iron overload, chronic HCV infection may have acted as a clinically relevant “second hit,” tipping the balance toward clinical PCT. UROD activity was not directly measured in this patient, and this mechanism therefore remains inferred rather than directly demonstrated.

Additional cofactors may also have contributed to disease expression. The patient’s occupational exposure to asphalt, a source of polycyclic aromatic hydrocarbons, may have induced cytochrome P450 (CYP1A2) activity, generating porphyrin intermediates and intensifying photosensitivity (5, 6). The coexistence of Gilbert’s syndrome (UGT1A1 –3,275 T > G) might have modestly reduced hepatic conjugation capacity, aggravating oxidative stress. These environmental and metabolic factors likely interacted synergistically rather than independently.

Following this mechanistic framework, SEC23B-related ER dysfunction may also influence hepatic stress responses. This potential interaction with chronic HCV infection remains speculative and cannot be confirmed in the current single case.

Clinically, the patient’s rapid improvement after direct-acting antiviral therapy underscores the value of addressing reversible triggers. Clearance of HCV normalized hepatic function, reduced ferritin levels, and completely resolved skin lesions, supporting the central role of viral and iron-driven oxidative mechanisms in this presentation. Ongoing monitoring remains essential, as residual iron overload and bilirubin metabolism variants may predispose to hepatic fibrosis or gallstone formation even after viral eradication (7). Direct hepatic iron quantification by MRI or histologic assessment was not available in this case.

Overall, this case supports a multifactorial model in which SEC23B mutations, chronic hemolysis, HCV infection, and environmental exposures jointly disturb iron homeostasis and hepatic redox balance, leading to secondary inhibition of UROD and manifestation of PCT. Recognizing such overlapping pathways can improve diagnostic accuracy and inform personalized management for patients with rare hematologic disorders complicated by metabolic or infectious triggers.

## Data Availability

The raw data supporting the conclusions of this article will be made available by the authors, without undue reservation.

## References

[1] Iolascon A Andolfo I Russo R. Congenital dyserythropoietic anemias. Blood. (2020) 136:1274–83. doi: 10.1182/blood.2019000948, 32702750

[2] Khoriaty R Vasievich MP Ginsburg D. The COPII pathway and hematologic disease. Blood. (2012) 120:31–8. doi: 10.1182/blood-2012-01-292086, 22586181 PMC3390960

[3] To-Figueras J. Association between hepatitis C virus and porphyria cutanea tarda. Mol Genet Metab. (2019) 128:282–7. doi: 10.1016/j.ymgme.2019.05.003, 31097365

[4] Rudnick S Bonkovsky HL. Editorial: hepatitis C and porphyria cutanea tarda in 2020. Aliment Pharmacol Ther. (2020) 51:1432–4. doi: 10.1111/apt.15728, 32445530

[5] Singal AK. Porphyria cutanea tarda: Recent update. Mol Genet Metab. (2019) 128:271–81. doi: 10.1016/j.ymgme.2019.01.004, 30683557

[6] Quansah R Cooper CJ Said S Bizet J Paez D Hernandez GT. Hepatitis C- and HIV-induced porphyria cutanea tarda. Am J Case Rep. (2014) 15:35–40. doi: 10.12659/AJCR.889955, 24470839 PMC3901625

[7] Heimpel H Anselstetter V Chrobak L Denecke J Einsiedler B Gallmeier K . Congenital dyserythropoietic anemia type II: epidemiology, clinical appearance, and prognosis based on long-term observation. Blood. (2003) 102:4576–81. doi: 10.1182/blood-2003-02-0613, 12933587

[8] Perrotta S del Giudice EM Carbone R Servedio V Schettini F Jr Nobili B . Gilbert's syndrome accounts for the phenotypic variability of congenital dyserythropoietic anemia type II (CDA-II). J Pediatr. (2000) 136:556–9. doi: 10.1016/S0022-3476(00)90026-X, 10753261

[9] Jang W Ha DJ Nahm CH Park J Kim SJ Lee JE . Identification of a novel splice variant in SEC23B gene in a patient with concomitant presence of congenital dyserythropoietic anemia II and Gilbert's syndrome. Hematology. (2024) 29:2343163. doi: 10.1080/16078454.2024.2343163, 38655690

