# Supplementary figures and images for: Case Report: HCV-triggered porphyria cutanea tarda in a patient with SEC23B-mutated congenital dyserythropoietic anemia type II
Source: Front Med (Lausanne). 2026 Jul 13;13:1817500. doi: 10.3389/fmed.2026.1817500 (PMC13402175; doi:10.3389/fmed.2026.1817500)

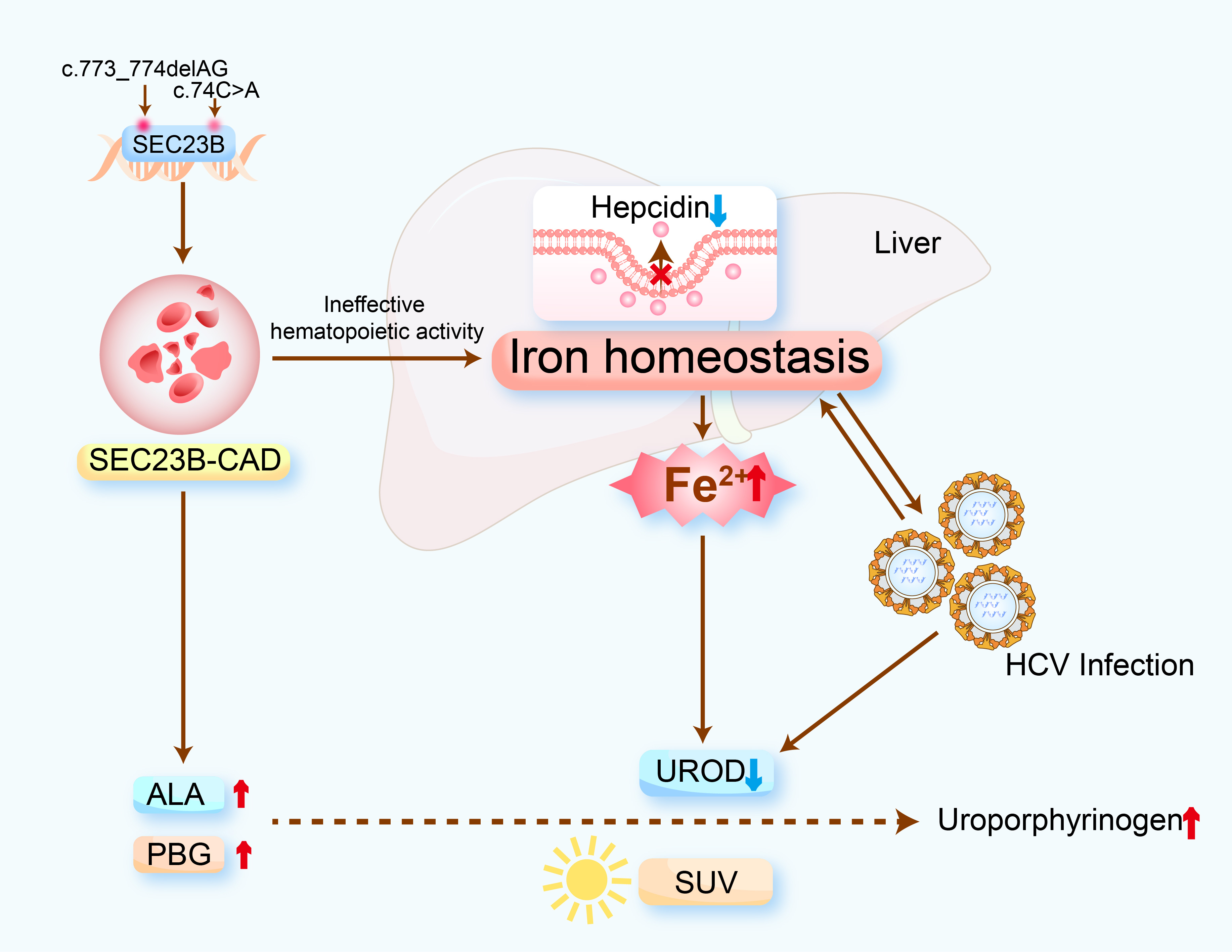

Supplement: Supplementary file 1 [file Image_1.jpg]
